# Androgen-Deprivation Therapy and Cardiovascular Disease Risk – The Role of Exercise in Prostate Cancer Treatment

**DOI:** 10.3389/fonc.2016.00200

**Published:** 2016-09-19

**Authors:** Bradley Wall

**Affiliations:** ^1^School of Psychology and Exercise Science, Murdoch University, Murdoch, WA, Australia

**Keywords:** prostate cancer, exercise, androgen-deprivation therapy, cardiovascular disease

Reduced levels of physical activity and increased levels of fatigue are commonly reported in prostate cancer patients treated with androgen-deprivation therapy (ADT) (1) that in turn reduces functional capacity. Reductions are seen in cardiorespiratory endurance, upper and lower body strength and endurance, and physical components of quality of life (1–3), which lead to inhibiting activities of daily living. In addition to these well-established side effects, cardiovascular disease (CVD) risk is now being increasingly associated with ADT (4, 5). Keating et al. (6) report ADT use is associated with higher risks of incident diabetes, coronary heart disease, acute myocardial infarction, and sudden cardiac death. The increasing body of literature supports an earlier report indicating that CVD is the most common form of mortality in men with prostate cancer, and not the actual cancer itself (7).

Exercise has been shown to be effective for improving surgical outcomes, reducing symptom experience, managing side effects, improving psychological health, maintaining physical function, and reducing fat gain and muscle and bone loss in cancer patients (8) and hence has the potential to reduce CVD risk factors. Studies in the past have used aerobic exercise (9), resistance exercise (9, 10), or a combination of aerobic and resistance exercise (11). Exercise programs have also differed in method of delivery with some home based (12) while others are group based in a clinic setting (11, 13) making comparisons as to the best treatment mode of exercise difficult.

## Resistance Training

Resistance exercise improves muscle strength and function and has been shown to be an effective intervention against sarcopenia (14). This exercise mode also leads to improvements in functional capacity and quality of life while reducing disability in individuals with and without CVD (15, 16). In prostate cancer patients undergoing treatment, studies have shown positive effects of resistance exercise on reducing musculoskeletal treatment side effects, decreasing fatigue, and improving quality of life (10, 13), despite a compromised hormonal profile (17). Segal and colleagues (10) had patients complete resistance exercises three times per week for 12 weeks and reported upper and lower body muscular fitness improvements of 42 and 32%, respectively, whereas Galvao et al. (13) had patients undergo resistance exercise two times per week for 20 weeks and reported significant improvements in upper body muscular strength (chest press 40% and seated row 42%), lower body strength (leg press 96%), functional performance (400 m walk 7.4%, stair climb 10.4%, and chair rise 27%), and balance (7.8%).

Improvements in muscle mass, such as those seen by Galvao et al. (11), are important not only in terms of mobility and functional performance but also in terms of assisting glucose disposal (18). Muscle stimulates insulin sensitivity and accounts for up to 80% of insulin-dependent glucose uptake (19). Numerous clinical studies have shown resistance training to lower the percentage of glycated hemoglobin and increase glucose disposal as well as favorably impact CVD risk factors in elderly individuals (20, 21). Srikanthan and Karlamangla (22) conducted a cross-sectional analysis of the National Health and Nutritional Examination Survey III data to investigate the possible correlation between relative muscle mass and insulin resistance and prediabetes. After adjusting for age, ethnicity, sex, and generalized and central obesity, the authors found for every 10% increase in skeletal muscle index (skeletal muscle mass relative to total body mass) was associated with an 11% reduction in insulin resistance and a 12% relative reduction in prediabetes. This correlation was stronger in non-diabetic patients (22) and was not just limited to the lower, sarcopenic end of the muscle mass distribution in the population, suggesting that increases in muscle mass, even above average levels, was associated with additional protection against insulin resistance and diabetes. Given the association of insulin resistance and CVD, the metabolic effect of muscle mass gained as a result of resistance training has the ability to alter CVD risk (23), particularly in an ADT-treated population where reductions in muscle mass have previously been reported (24).

## Aerobic Training

Studies employing aerobic training only interventions have primarily utilized breast cancer patients, with promising outcomes reported in physical function, fatigue mitigation, and quality of life (25–27). Of the aerobic intervention studies in prostate cancer patients, radiotherapy has been the most common treatment modality (28, 29) with one study utilizing ADT-treated prostate cancer patients (9). Segal et al. (9) reported beneficial effects of the aerobic training for both fatigue mitigation and maintenance of aerobic fitness.

With regard to non-cancer patients, it is well established that cardiorespiratory fitness attenuates the mortality risk associated with metabolic syndrome in healthy men, independent of body mass (30), indicating that cardiorespiratory fitness is of greater importance than body mass *per se*. High cardiorespiratory fitness levels have also been found to attenuate the increased arterial stiffness in patients with metabolic syndrome. Increases in arterial stiffness, independent of age and blood pressure, have been reported in men receiving ADT (31–33) highlighting the need for prescribed aerobic exercise programs focused on improving cardiorespiratory fitness to offset the metabolic related treatment toxicities in ADT-treated patients.

## Combined Aerobic and Resistance Training

Although there are studies that have investigated the effects that resistance training programs and aerobic training programs have on prostate cancer patients undergoing treatment, very few have utilized a combined aerobic and resistance training intervention with prostate cancer patients undergoing ADT. Culos-Reed et al. (12) were one of the first study to attempt to employ a combined aerobic and resistance training program in ADT-treated prostate cancer patients; however, the study was a low-intensity home-based intervention and hence lacked the control of a laboratory-based intervention. This apparent lack of control may have been the reason for a lack of statistically significant differences. Galvao et al. (11) were the first group to conduct a clinic-based supervised, randomized controlled study to evaluate the combined effects of a resistance and low volume aerobic exercise program in men undergoing ADT. Galvao et al. (11) reported favorable changes in total body and regional lean mass as well as improvements in muscular strength and functional performance outcomes. Cardiorespiratory capacity only showed borderline improvement, but this was thought to be a result of the low level of aerobic exercise prescribed. Participants undertook 30–40 min of aerobic exercise per week, which is well below 150 min recommended by the American Heart Association for good health. However, the promising aspect of this study was that despite the low dose of aerobic exercise prescribed, borderline changes were still seen in cardiorespiratory capacity.

## Conclusion

Given the recent attention that ADT and the associated CVD risk have received, it is clear that exercise interventions specifically targeting CVD risk outcomes are required. Previous studies have targeted resistance training or aerobic training alone and have reported positive outcomes specific to the training modality. What is now needed are exercise interventions that apply sound aerobic and resistance training principles in combination to prevent the development of CVD in men undergoing ADT for the treatment of prostate cancer.

## Author Contributions

BW is responsible for all aspects of this opinion piece.

## Conflict of Interest Statement

The author declares that the research was conducted in the absence of any commercial or financial relationships that could be construed as a potential conflict of interest.
